# Instrument development and validation of the stroke pre-hospital delay behavior intention scale in a Chinese urban population

**DOI:** 10.1186/s12955-014-0170-8

**Published:** 2014-11-29

**Authors:** Qiuli Zhao, Li Yang, Qingqing Zuo, Xuemei Zhu, Xiao Zhang, Yanni Wu, Liu Yang, Wei Gao, Minghui Li

**Affiliations:** School of Nursing, The 2nd Affiliated Hospital of Harbin Medical University, Harbin Medical University, 246 Xuefu Road, Harbin, HeiLongJiang Province 150086 China; Department of Nephrology, The 2nd Affiliated Hospital of Harbin Medical University, Harbin Medical University, Harbin, HeiLongJiang Province 150086 China

**Keywords:** Pre-hospital delay, Behavioral intention, High-risk stroke patients

## Abstract

**Background:**

Several stroke impairment scales are currently available for stroke patients but none of them provide information regarding the pre-stroke behavioral intentions of high-risk stroke patients and their relatives. This study’s objective was to generate and validate a new written tool, the Stroke Pre-hospital Delay Behavior Intention (SPDBI) scale. It is suitable for use with high-risk stroke patients and their relatives to predict the likelihood of pre-hospital delay.

**Methods:**

From a review of related studies, we formulated a prototype scale. We interviewed ten stroke patients in a semi-structured iterative process that included interviews with experts, high-risk patients, and their family members. Then, we pretested and filtered items. We next used a large sample size and factor analysis to determine the scale’s structure. Finally, we checked the reliability and validity of the scale.

**Results:**

We identified five sub-domains (stroke warning signs, non-treatment justification, symptom attributions, habitual response style, and emergency system use). The SPDBI demonstrated good internal consistency and test-retest reliability (Cronbach’s α =0.808; Intraclass Correlation Coefficient [ICC] =0.797).

**Conclusions:**

This SPDBI scale is a reliable, and valid measure of the likeliness of pre-hospital delay in high-risk stroke patients and their family members. It may provide scientific assessment for targeted health education intervention.

**Electronic supplementary material:**

The online version of this article (doi:10.1186/s12955-014-0170-8) contains supplementary material, which is available to authorized users.

## Background

Stroke is the third leading cause of death in the United States, Canada, Europe, and Japan [1]. In China, cerebrovascular disease has become the leading cause of mortality and morbidity in both urban and suburban populations with 1,500,000–2,000,000 new strokes each year [1]. Many studies have demonstrated that thrombolytic therapy is only effective within the first 4.5 hours after the onset of an ischemic stroke [2,3]. Early intensive blood pressure-lowering treatment is clinically feasible, well tolerated, and appears to reduce hematoma growth in acute intracerebral hemorrhage (ICH) [4]. People need to access emergency medical services quickly when early stroke symptoms occur. However, in most communities, only 1–7% of stroke victims arrive at the hospital in time for stroke revascularization therapies [1]. Moreover, in China, only 37% of patients were sent to the hospital within the effective therapeutic time window for strokes, and the rate of thrombolytic therapy for an ischemic stroke is estimated at only 1–3% [5]. In addition, more than 80% of Chinese stroke patients suffer their first stroke symptoms at home [5].

Pre-hospital delay time is the time from symptom onset to hospital arrival [6]. The delay occurs for numerous reasons; for instance, many patients and their relatives do not think their symptoms are serious, and a wait-and-see approach is adopted before seeking treatment. A serious stroke makes patients unable to ask for help themselves [7]. These pre-hospital delay behavioral intentions increase pre-hospital delay time and decrease the therapeutic efficacy of patient interventions.

The stroke impairment scales with warning signs were developed to quickly assess stroke patients by the public and/or paramedics, and include scales such as the Cincinnati Pre-Hospital Stroke Scale (CPSS), the Los Angeles Pre-hospital Stroke Screen (LAPSS), and the Face Arm Speech Test (FAST) [8-10]. These scales contain items about stroke symptoms, but simple recognition of specific stroke symptoms may not be associated with reduced pre-hospital delay [11,12]. Additionally, the Stroke Action Test (STAT) [13] evaluates the public’s reaction to a stroke. The scores of the STAT predict the actions that the public would take if stroke symptoms occur, but cannot specifically ascertain the pre-hospital delay in behavioral intention.

Stroke pre-hospital delay time has increasingly become the focus of researchers’ attention. Most studies have focused on determining time from patient stroke onset to hospital registration, and then judging whether there is a delay [14-17]. However, they have not addressed decreasing that delay. New tools capable of detecting the participant’s behavioral intention if stroke symptoms occur and then assessing the possibility of a pre-hospital delay are needed.

Therefore, we developed a scale named the Stroke Pre-Hospital Delay Behavior Intention (SPDBI) scale, which includes not only stroke warning signs but also specific items on how the participants would think about or react to stroke signs in order to reduce stroke pre-hospital delays. It is intended to provide a scientific assessment tool for targeted health education intervention.

## Methods

The study protocol was approved by the ethics committee of the 2nd Affiliated Hospital of Harbin Medical University. All of the experts and participants signed the informed consent form, were provided with a verbal explanation about the purpose of the study, and informed that participation was voluntary, confidential, and anonymous.

### Samples

Using convenience sampling, we advertised for and telephoned participants who agreed to participate in this study from the 2nd Affiliated Hospital of Harbin Medical University and local community groups in Harbin City, China. The criteria for the participants were (1) high-risk stroke patients with a confirmed diagnosis of diabetes, coronary artery disease, hypertension, or hyperlipidemia; or (2) family members or those who might be responding to the stroke symptoms (e.g., partners, adult children, other family members, friends, or caregivers. Participants were volunteers 18 years of age or older. Participants were excluded if they had any of the following characteristics: (1) history of stroke, dementia, or severe psychiatric disorders; (2) altered consciousness; (3) deafness or blindness; or (4) were medical practitioners. Participants were in the hospital unit or invited to visit the community service center to complete the scale, which was accompanied by a short questionnaire about their personal details.

Initially, we included 30 experts for the preliminary scale. The expert criteria were medical doctors or nurses who had been engaged in related work for more than 10 years (from 6 neurology departments, 3 neurosurgery departments, and 1 emergency department). Then, 20 stroke high-risk patients and their family members who met the patient criteria were also invited from a geriatric department. A total of 312 eligible participants (high-risk stroke patients and their family members) were then drawn from 6 settings (1 gerontology department, 1 cardiovascular medicine department, 1 endocrinology department, and 3 local community groups) for item analysis.

The revised temporary scales were assessed in 616 eligible new participants (high-risk stroke patients and their family members) from 10 locations (2 gerontology departments, 2 cardiovascular medicine departments, 2 endocrinology departments, and 4 local community groups). The test-retest reliability was assessed by comparing the SPDBI scores of 86 additional participants (high-risk stroke patients and their family members) from 5 local community groups.

### Procedure

**Figure 1:**
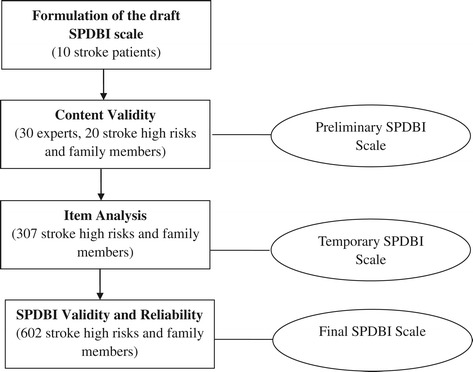
**Schematic representation of the Stroke Pre-hospital Delay Behavior Intention (SPDBI) scale validation process.**

A schematic representation of the study design is shown in Figure 1. The final SPDBI has two response formats depending on item phrasing. Both formats are based on a 5-point Likert scale of 1 (*strongly serious or disagree*) to 5 (*nothing serious or highly agree*). The overall SPDBI score is calculated by adding the item scores together. A higher score indicates a greater likelihood of pre-hospital delay.

### Stage 1: formulation of the draft SPDBI scale

From a review of stroke scales, the Chinese guidelines for management and treatment of acute ischemic stroke (2010) [18], public education materials on strokes, and pre-hospital delay factors found in related studies, we formulated a prototype scale. We next verified the prototype by conducting semi-structured interviews in ten stroke patients. We asked them “what symptoms appeared when the stroke occurred, what did you think, and what did you do?” We recorded their answers and developed new items from them. Then, we hypothesized and developed a 63-item, 6-factor (Factor 1: stroke warning signs, Factor 2: barriers to going to a doctor, Factor 3: symptom attributions, Factor 4: habitual response style, Factor 5: hospital chosen, Factor 6: vehicle use) draft SPDBI scale. All of the items were presented in lay language so that the participants could clearly understand the meaning [13].

### Stage 2: content validity

Two surveys were sent to experts. The first survey included an introductory letter, written consent forms, a questionnaire about the draft SPDBI scale, and the inquiry deadline. The authors visited the various clinical departments and personally disseminated the first survey questionnaire to the experts who met the recruitment requirements. A week later, the authors received the first survey results and analyzed them. Two weeks after dissemination the second survey began. This second survey included a summary of experts’ advice from the first survey and the content validity value judgment—the correlation between item and the stroke pre-hospital delay behavior intention, from 4 (*strongly related*) to 1 (*not related*). The delivery method was the same as the first round. After the two surveys, 20 participants were asked to complete the preliminary scale to test the scale’s comprehension and wording.

### Stage 3: item analysis

Items in the preliminary scale that satisfied two or more of the following conditions were modified to formulate a temporary scale. (1) The variable’s standard deviation was less than 0.75. (2) The degree of high-score and low-score group differentiation had no statistical significance at *p* >0.05. (3) Response analysis showed three or more (>2) ratings of the response rate that were less than 10%. (4) Internal consistency analysis showed an item and total correlation coefficient score of less than 0.4. The remaining items were assessed by principal component factor analysis with varimax rotation to delete items that loaded <0.4 or cross-loaded (loaded on ≥2 factors with values ≥0.4 and with a difference of <0.2 between them) [19].

### Stage 4: SPDBI reliability and validity

A reliability and validity study was then performed.

### Statistical analysis

#### Characteristics of the sample

Descriptive statistics were used to analyze demographic information.

### Content validity

Item content validity index in stage 2 is the number of expert choices of 3 and 4 divided by the total number of experts. Total content validity index (CVI) is the average of the item content validity index.

### Exploratory factor analysis (EFA)

Principal Components Analysis (PCA) and Parallel analysis [20] were conducted. Parallel analysis is often recommended as the best method to assess the true number of factors [21].To assess the suitability of the factor solution, Bartlett’s test of sphericity [22] should be significant, and the Kaiser-Meyer-Olkin (KMO) measure of sampling adequacy should be >0.6 [23]. The number of factors met the decision rules of (1) Kaiser’s criterion (eigenvalues >1.0) [24] (2) inspection of the scree plot [25], and (3) Parallel analysis: Eigenvalues obtained from PCA with eigenvalues exceeding the values obtained from the corresponding random data set are retained [23],we conducted the parallel analysis online (http://ires.ku.edu/~smishra/parallelengine.htm).

### Confirmatory factory analysis (CFA)

Confirmatory factor analysis (CFA) on the structural model was also performed in Stage 4; analysis of moment structures (Amos, 17.0) was used to test the model. The models’ goodness of fit was evaluated using absolute and relative indices [26], including the root mean squared error of approximation (RMSEA; <0.08 acceptable), adjusted goodness of fit index (AGFI; >0.90 acceptable), Tucker-Lewis index (TLI), comparative fit index (CFI), and goodness of fit index (GFI) all >0.90 acceptable.

### Discriminate validity

The assessment of discriminate validity was exploratory.The SPDBI scale total scores were compared among different groups. For the comparison between two groups, the *t*-test was used. One-way analysis of variance (ANOVA) for multiple comparisons was used where more than 2 groups were available and the data followed a normal distribution.

### Internal consistency reliability and test-retest reliability

Nunnaly’s criterion for satisfactory internal consistency reliability is a Cronbach’s alpha of ≥0.7 [27]. We also measured intraclass correlation coefficient (ICC) according to the following values: weak correlation (≤0.4), moderate correlation (0.41–0.60), good correlation (0.61–0.80), and excellent correlation (0.81–1.00) [28].

## Results

### Characteristics of the sample

Experts’ mean age was 39.5 years (±7.2, range =30–52). Over half had 10–20 years of experience (70%) and the others had more than 20 years of experience (30%). Approximately half had the professional title of nurse- or doctor-in-charge (43.3%). Level of education was divided roughly equally between doctoral level (33.3%), master’s degrees (36.7%), and bachelor’s degrees (30%). All were involved in work related to stroke pre-hospital delay: neurology (70%), neurosurgery (20%), and emergency departments (10%), see Table 1. Two experts were excluded in the second survey because of a business trip.

**Table 1:** **Demographic characteristics of the first survey content validity sample**

**Characteristics**	**%**	**n**
Age,		
<45	73.3	22
45–59	26.7	8
Years of experience		
10–20	70.0	21
>20	30.0	9
Professional title		
Senior nurse	3.3	1
Nurse-in-charge/doctor-in-charge	43.3	13
Co-chief nurse/associate doctor	23.3	7
Chief nurse/chief physician	30.0	9
Highest education completed		
Bachelor	30.0	9
Master	36.7	11
Doctor	33.3	10
Professional field		
Neurology	70.0	21
Neurosurgery	20.0	6
Emergency department	10.0	3

Five participants were excluded for various reasons in stage 3, including participation refusal (n =2), worsening patient condition (n =2), and incomplete information (n =1), therefore, a total of 307 participants were included in item analysis. Similarly, fourteen participants were excluded in Stage 4 for different reasons, including lack of information (*n* =4), refusal to participate (n =3), worsening patient condition (*n* =4), and other reasons (*n =*3). The remaining 602 participants (Table 2) were included in the final validity and reliability analysis (282 men and 320 women; 320 high-risk stroke patients and 282 family members; mean age 48.29 years [±13.91]).

**Table 2:** **Demographic characteristics of Stage4 sample and comparison of Mean SPDBI Scores by 602 participants**

**Characteristics**	**N(%)**	**Mean(SD)**	**P value**	**95% CI**
**Gender**				
Male	282(46.8%)	81.34(14.94)	0.596	−3.15-1.73
Female	320(53.2%)	82.05(15.48)		
**Age**				
18-85	602(100%)	81.72(15.22)		
<40	171(28.4%)	80.99(14.16)	0.242	
40-60	327(54.3%)	82.64(15.08)		
>60	104(17.3%)	80.04(17.17)		
**Habitual residence**				
City center	445(73.9%)	80.94(15.75)*	P < 0.01	1.62-8.50
Suburb	67(11.1%)	81.16(13.75)		
Country	90(15.0%)	86.00(12.87)*		
**Classification**				
High-risk stroke patient	320(53.26%)	81.31(15.98)	0.480	−3.32-1.56
Family member	282(46.8%)	82.19(14.33)		
**Family per capita monthly income (yuan)**				
<1000	67(11.1%)	83.22(13.92)	P < 0.05	0.57-5.84
1000–2000	212(35.2%)	83.47(15.35)*		
>2000	323 (53.7%)	80.26(15.29)*		
**Physical examination**				
More than once per year	195(32.4%)	80.11(17.25)*	P < 0.05	0.27-6.32
A few years at a time	206(34.2%)	83.36(14.53)*		
Never	201(33.4%)	81.61(13.64)		
**Self-report stroke knowledge**				
Received stroke knowledge education	76(12.6%)	78.92(18.96)*	P < 0.05	0.28-8.41
Saw someone suffering from stroke	289(48.0%)	81.30(15.27)		
Know nothing about stroke	237(39.4%)	83.13(13.66)*		

Notes: N = 602. There are statistically significant differences between the groups with “*”.

### Content validity

Data from the expert survey indicated that the content validity index (CVI) was 0.901. After the experts reached consensus, the preliminary SPDBI scale had 60 items and data showed that all 20 participants (10 high-risk stroke patients and 10 relatives) agreed that it was easy to understand with no difficulties reported.

### Item analysis

After item analysis in stage 3, we kept 45 items. After the preliminary factor analysis on the test results, there were 7 items removed, formulating the 38-item temporary SPDBI scale.

### Exploratory factor analysis (EFA)

The KMO test (0.897) showed adequate sampling adequacy, and Bartlett’s test was significant (*df* =703, *p* =0.000). Preliminary analyses produced a seven-factor model. A total of 11 items with a load <0.4 or with cross-load were deleted, PCA were finally revealed 5 factors, which was also supported by parallel analysis (Table 3). 27 items loaded substantially onto these 5 factors. The final produced factors (Table 4) included Factor 1, stroke warning signs (9 items) by which participants judge the severity of the stroke symptoms listed; Factor 2, non-treatment justification (8 items )describing alternative explanations and not receiving treatment; Factor 3, symptom attributions (4 items) or participants’ analyses of the stroke symptom causes; Factor 4, habitual response style (3 items) to determine whether the participants usually responded to medical symptoms immediately; and Factor 5, emergency system use (3 items) regarding the likelihood of the participant’s choice of hospitalization and transport. The factor scree plot also showed a suitable 5-factor solution; these factors contributed to 53.447% of the variance.

**Table 3:** **The results of parallel analysis**

**Eigen values**	**Means**	**Percentile**
7.479	1.511189	1.569466
4.484	1.445252	1.486443
2.169	1.405245	1.437552
1.745	1.366892	1.395265
1.343	1.332469	1.357914

**Table 4:** **Factor structure of the final version of SPDBI**

**Sub-domains and items**	**Item loadings and Variance**
**Sub-domain 1: stroke warning signs**	
V1.Inconsistent in thinking and language; answers to the problems such as time and place are unclear; restlessness	0.777
V2.When asleep, intense stimulation is required to wake up; answers are irrelevant or vague; when stimulation is stopped, fall asleep quickly	0.737
V3.Can be awakened and was able to answer simple questions, but slowly, then continued to sleep when stimulation stopped	0.737
V4.Weakness, heaviness, or numbness on one side of the limb	0.722
V5.Vertigo (see rotation ), blacked out	0.720
V6.Severe headache, vomiting, neck stiffness, neck pain	0.685
V7.Double vision on one side of the eyes	0.673
V8.Clear pronunciation, but of incorrect and ambiguous words	0.658
V9.Blurred vision on one side of the eyes	0.624
**Sub-domain 2: non-treatment justification**	
V10.Don’t go to the hospital because the results are the same whether or not you go	0.793
V11.Don’t go to the hospital because it is too much trouble	0.783
V12.Don’t go to the hospital because worried about added burden to family.	0.780
V13.Don’t go to the hospital because symptoms are from being old and weak	0.762
V14.Don’t go to the hospital because body is usually ok and symptoms are no big deal.	0.754
V15.Patient will soon recover and symptoms are nothing important	0.720
V16.Patient will first rest and see how they feel since the weather is bad	0.641
V17.I will wait since there is no one around to help me	0.613
**Sub-domain 3: symptom attributions**	
V18.Sudden weakness, heaviness, or numbness on one side of the limb is just recent tiredness	0.671
V19.Sudden blurred vision in one or both eyes is from excessive eye use	0.661
V20.Weakness, clumsiness on one side of the limb in the morning, because pressure to stay in bed	0.644
V21.Sudden headache and dizziness are caused by a cold	0.530
**Sub-domain 4: habitual response style**	
V22.My first thought is to have a rest at onset of symptoms	0.692
V23.My first thought is to take some medicine at onset of symptoms	0.768
V24.If my symptoms don’t improve(or worsen),then I will go to the hospital	0.613
**Sub-domain 5: emergency system use**	
V25.Don’t call an ambulance because of the high cost	0.721
V26.I can’t think to call an ambulance at first	0.683
V27.I chose a Chinese medicine hospital suggested by an acquaintance	0.606
**Total scale**	**53.447%**

### Confirmatory factory analysis (CFA)

Table 5 shows that the degree of fit and stability of the structural model are good to excellent.

**Table 5:** **Fit indices for the model**

	***x*** ^**2**^ **/df**	**GFI**	**AGFI**	**RMSEA**	**CFI**	**TLI**
Model	2.286	0.917	0.900	0.046	0.924	0.915
Fit criteria	<5	>0.9	>0.8	<0.08	>0.9	>0.9

GFI, goodness-of-fit index; AGFI, adjusted goodness-of-fit index; RMSEA, root mean square error of approximation; CFI, comparative fit index; TLI, Tucker-Lewis index.

### Discriminate validity

The SPDBI’s total score had statistically significant differences in the following aspects: habitual residence, family per capita monthly income, physical examination, and self-report stroke knowledge (*p* <0.05) (Table 2). However, age, classification, and gender (Table 2) were not associated with the total score.

### Internal consistency reliability and test-retest reliability

The Cronbach’s alpha is 0.808. Test-retest reliability for total scale was good with ICC =0.797, and for sub-domains ICC = 0.676-0.819(Table 6).

**Table 6:** **The SPDBI ICCs across the two weeks of the test-retest interval**

**Sub-domains**	**Number of items**	**ICC**	**95% CI**
Sub-domain 1	9	0.777	0.658-0.855
Sub-domain 2	8	0.819	0.722-0.882
Sub-domain 3	4	0.742	0.605-0.832
Sub-domain 4	3	0.714	0.562-0.814
Sub-domain 5	3	0.676	0.503-0.789
Total	27	0.797	0.688-0.867

## Discussion

Behavioral intention refers to the subjective probability of an individual engaging in a particular behavior and reflects the will an individual adopts for a particular behavior [29]. Our research defines pre-hospital delay in behavioral intention as delayed treatment from symptom appearance to reaching a hospital with appropriate interventions. We developed a scale that includes not only the severity of stroke warning signs but also specific items on how the participants would think about or react to stroke signs. The SPDBI may indeed be particularly suitable for use in behavior intention programs, which recognize the potential impact of participants’ cognition and behavior on stroke pre-hospital delay. First, we can determine which predictors of the scale are more likely to lead to participants’ pre-hospital delay. Second, we could use this tool to select those at high risk of stroke and their family members who scored higher, and provide them with systematic health education intervention. The more targeted such health education is, the more likely participants are to cooperate and save social resources at the same time. Third, by applying this scale before and after health education, we can compare the scores before and after the intervention with the pre-hospital delay time length or rate of thrombolysis, using tracing methods to verify the exactness of the scale prediction.

The SPDBI scale has good reliability and validity. We involved experts in neurology, neurosurgery, and emergency departments to test the content validity. All of these experts are in close contact with stroke patients, and communicate with patients in the process of admits, treatment, or care, so they have significant understanding of the degree of pre-hospital delay and factors influencing patients. In addition, the experts were distributed among different units and did not know the names of other experts and their departments, so we avoided communication between experts. More importantly, we also selected 20 high-risk participants and their close family members to test the wording and understanding. Comprehensively, the content validity of this research is good and, after further item revision, potentially improvable. Both exploratory and confirmatory factor analyses are appropriately used when a hypothesized measurement model is evaluated [30]. The sample size should be at least 10–15 individuals per variable for an exploratory factor analysis [30]. Our sample size was large enough for the analyses. EFA shows 5 conceptually clear and psychometrically robust sub-domains and CFA suggests that the fit of the model’s structure and stability is good. Our result for Cronbach’s alpha is high. The stability of the total scale is good (ICC >0.7).

The discriminate validity evidence is that the scale can distinguish between groups of differences, the evidences are as follows:

First, the result showed that the group with poorer self-report stroke knowledge before testing had a higher SPDBI score than the group with better knowledge. As the SPDBI scale score predicted, we found similar results to other studies, i.e., knowing someone who had suffered a stroke was not associated with shorter pre-hospital delay, [31] and stroke knowledge received from other information sources was associated with shorter pre-hospital delay [32,33].

Second, the SPDBI scale could identify different total scores between groups with different family per capita monthly income and groups with different habitual residence. The groups with lower family per capita monthly income scored higher. This may be because although total public expenditure on health insurance in China has been rising steadily since 2006, the level of satisfaction with the health care system has remained low. There are several out-of-pocket medical costs [34], meaning that lower income people may be more likely to delay hospitalization. Besides, the groups with habitual residence in country scored higher, this may be because of greater distances from the hospital for treatment; ambulance arrival times remain varied across China [35] and EMS systems are absent in most Chinese areas [36].

Third, the participants who have fewer physical examinations will potentially delay hospitalization. One reason could be that people understand their health condition through physical examinations, which could help them to take timely and preventive actions. The other reason might be that doctors and nurses were regarded as the best source of stroke information [37], and people were encouraged to take a positive attitude when facing health problems in the process of physical examinations. Furthermore, physical examinations promoted access to health knowledge for people who prefer not to be subjected to medical checkups; this is especially true of the free physical examination by some work units in China.

Fourth, there were no significant difference in SPDBI subscale and total scores by gender, our analysis predicted gender was not a significant factor associated with pre-hospital delays in the presentation of acute stroke in urban China (Table 2). Jin et al. [5] found a similar result. However, data regarding gender differences in other countries for knowledge about stroke symptoms and correct behavior are conflicting. Women had better knowledge of stroke symptoms and faster arrival to the hospital in some studies [37,38], but poorer knowledge of stroke symptoms and later arrival to the hospital among older stroke patients [16], and were not associated with stroke presentation or management in another study [39].One explanation for our result might be the Chinese traditional culture of promoting family harmony. Most of our participants (93.2%), even the aged, live with their family members, thus reducing the possibility of older persons (especially older women) living alone, and are more likely to fall requiring help quickly [16]. Even so, we need to note that women have a greater risk of dying from stroke owing to their longer life expectancy [39].

We evaluated the instrument not only for use with groups of less healthy individuals (those with a high risk of stroke) but also their close family members. Important findings from other studies show that more than 80% [40] of patients had their strokes at home and these patients experienced longer delays. However, the person seeking medical help was rarely the patient himself. Patients only called an ambulance for themselves 3% of the time, frequently relying on family members instead. Indeed, in 37–68% of the cases, the decision (use of EMS) is made by a family member [5,40]. Any program aimed at increasing stroke awareness needs to target a broad community audience. Our study therefore highlights the urgent need to evaluate the high-risk stroke patients’ family members. The authors have compared the scores of high-risk patients and their family members, and an independent *t*-test found that their scores had no significant differences (p >0.05). Family members should also know the seriousness of stroke symptoms and the benefits of immediately going to a hospital for treatment if a stroke occurs.

The mean of the total scale score was 81.72 ± 15.22. If we consider this mean score as a cut-off point, then scores that are higher than this point predict a higher possibility of pre-hospital delay, and special attention would need to be paid to those in this group. In our results, 327 of 602 (54.3%) participants’ total scores surpassed the cut-off. This point is slightly lower than the 63% of stroke patients with pre-hospital delay reported by Jin et al. [5]. This may be because more than 80% of our participants are from cities where medical treatment is more convenient and the higher incidence of stroke in northeastern China may increase opportunities for health education.

### Limitations/future direction

There were some limitations in our study. First, we could not evaluate the psychometric properties of convergent validity, because we have not found other instruments that predict stroke pre-hospital delay. Furthermore, the scale is a Chinese version (Additional file 1). In China, people may face problems such as “high cost for an ambulance” or “prefer a Chinese medicine hospital,” which may not generalize to other countries. Further cross-cultural revisions and validation are needed in the international application of the scale in the future. Third, we need to continue working on ways to portray “warning sign” symptoms in the sub-domain more realistically, perhaps through the use of pictures or multimedia technology, as this would increase the predictive value of the scale. Finally, more than 80% (512/602) of our participants are citizens of Harbin and responses to acute strokes could not, therefore, be generalized to rural areas or remote regions of China. It would also be important to evaluate the instrument for use with individuals from those areas in a future study.

## Conclusion

In summary, the present study has rigorously developed and validated the SPDBI scale, providing scores with good reliability and validity. This scale assesses high-risk stroke patients and their family members’ possibility of pre-hospital delay if stroke happens, and might help to decrease stroke pre-hospital delay. Thus, it would greatly facilitate more targeted public education efforts in China.

## Additional file

Additional file 1:
**The English and Chinese version of SPDBI scale, and the electronic ethic prove.**
