# Supplementary material for: Instrument development and validation of the stroke pre-hospital delay behavior intention scale in a Chinese urban population
Source: Health Qual Life Outcomes. 2014 Nov 29;12:170. doi: 10.1186/s12955-014-0170-8 (PMC4264611; doi:10.1186/s12955-014-0170-8)
Supplement: Additional file 1: — The English and Chinese version of SPDBI scale, and the electronic ethic prove. [file 12955_2014_170_MOESM1_ESM.doc]

**Additional file 1**

**Appendix1: Stroke Pre-hospital Delay Behavior Intention Scale**

| No. | When you (or you find someone) in the following situations, you think: | Very serious | Serious | Generally serious | Not too serious | Nothing serious |
| --- | --- | --- | --- | --- | --- | --- |
| 1 | Inconsistent in thinking and language; answers to the problems such as time and place are unclear; restlessness | 1 | 2 | 3 | 4 | 5 |
| 2 | When asleep, intense stimulation is required to wake up; answers are irrelevant or vague; when stimulation is stopped, fall asleep quickly | 1 | 2 | 3 | 4 | 5 |
| 3 | Can be awakened and was able to answer simple questions, but slowly, then continued to sleep when stimulation stopped | 1 | 2 | 3 | 4 | 5 |
| 4 | Weakness, heaviness, or numbness on one side of the limb | 1 | 2 | 3 | 4 | 5 |
| 5 | Vertigo (see rotation ), blacked out | 1 | 2 | 3 | 4 | 5 |
| 6 | Severe headache, vomiting, neck stiffness, neck pain | 1 | 2 | 3 | 4 | 5 |
| 7 | Double vision on one side of the eyes | 1 | 2 | 3 | 4 | 5 |
| 8 | Clear pronunciation, but of incorrect and ambiguous words | 1 | 2 | 3 | 4 | 5 |
| 9 | Blurred vision on one side of the eyes | 1 | 2 | 3 | 4 | 5 |
| No | If you observe the above symptoms, do you agree with the following ideas? | Agree very much | Agree | Don’t know | Agree less | Disagree |
| 10 | Don’t go to the hospital because the results are the same whether or not you go | 5 | 4 | 3 | 2 | 1 |
| 11 | Don't go to thehospital because it is too much trouble | 5 | 4 | 3 | 2 | 1 |
| 12 | Don't go to the hospital because worried about added burden to family. | 5 | 4 | 3 | 2 | 1 |
| 13 | Don't go to the hospital because symptoms are from being old and weak | 5 | 4 | 3 | 2 | 1 |
| 14 | Don't go to the hospital because body is usually ok and symptoms are no big deal. | 5 | 4 | 3 | 2 | 1 |
| 15 | Patient will soon recover and symptoms are nothing important | 5 | 4 | 3 | 2 | 1 |
| 16 | Patient will first rest and see how they feel since the weather is bad | 5 | 4 | 3 | 2 | 1 |
| 17 | I will wait since there is no one around to help me | 5 | 4 | 3 | 2 | 1 |
| 18 | Sudden weakness, heaviness, or numbness on one side of the limb is just recent tiredness | 5 | 4 | 3 | 2 | 1 |
| 19 | Sudden blurred vision in one or both eyes is from excessive eye use | 5 | 4 | 3 | 2 | 1 |
| 20 | Weakness, clumsiness on one side of the limb in the morning, because pressure to stay in bed | 5 | 4 | 3 | 2 | 1 |
| 21 | Sudden headache and dizziness are caused by a cold | 5 | 4 | 3 | 2 | 1 |
| 22 | My first thought is to have a rest at onset of symptoms | 5 | 4 | 3 | 2 | 1 |
| 23 | My first thought is to take some medicine at onset of symptoms | 5 | 4 | 3 | 2 | 1 |
| 24 | If my symptoms don't improve(or worsen),then I will go to the hospital | 5 | 4 | 3 | 2 | 1 |
| 25 | Don't call an ambulance because of the high cost | 5 | 4 | 3 | 2 | 1 |
| 26 | I can’t think to call an ambulance at first | 5 | 4 | 3 | 2 | 1 |
| 27 | I chose a Chinese medicine hospital suggested by an acquaintance | 5 | 4 | 3 | 2 | 1 |

**附件1：脑卒中院前延迟行为意向测评量表**

| **序号** | **当您（或您发现有人）出现下列情况时，您认为：** | **非常**  **严重** | **比较**  **严重** | **一般** | **不太**  **严重** | **不**  **严重** |
| --- | --- | --- | --- | --- | --- | --- |
| 1 | 思维和语言不连贯，对时间、地点等问题的回答不清，躁动不安 | 1 | 2 | 3 | 4 | 5 |
| 2 | 处于熟睡状态，能被摇动身体等强烈刺激叫醒，醒后答话含糊或答非所问，停止刺激很快入睡 | 1 | 2 | 3 | 4 | 5 |
| 3 | 睡眠时间延长，能被叫醒，且能回答简单的问题，但反应迟钝，停止刺激又继续入睡 | 1 | 2 | 3 | 4 | 5 |
| 4 | 一侧肢体无力、沉重或麻木 | 1 | 2 | 3 | 4 | 5 |
| 5 | 眩晕（视物旋转）、眼前发黑 | 1 | 2 | 3 | 4 | 5 |
| 6 | 既往少见的严重头痛、呕吐，伴有颈部僵硬，颈背痛 | 1 | 2 | 3 | 4 | 5 |
| 7 | 一侧眼睛视物双影 | 1 | 2 | 3 | 4 | 5 |
| 8 | 说话发音清楚，用词不正确，词不达意 | 1 | 2 | 3 | 4 | 5 |
| 9 | 一侧或双眼视力模糊 | 1 | 2 | 3 | 4 | 5 |
| **序号** | **当您出现上述情况时，您是否赞同以下观点？** | **非常赞同** | **比较赞同** | **不**  **确定** | **不太赞同** | **不**  **赞同** |
| 10 | 我这病去不去医院一个样，不去医院 | 5 | 4 | 3 | 2 | 1 |
| 11 | 去医院太麻烦，不去医院 | 5 | 4 | 3 | 2 | 1 |
| 12 | 担心给家人增添负担，不去医院 | 5 | 4 | 3 | 2 | 1 |
| 13 | 应该还是老毛病，不去医院 | 5 | 4 | 3 | 2 | 1 |
| 14 | 平时身体条件还行，不会有大事，不去医院 | 5 | 4 | 3 | 2 | 1 |
| 15 | 很快就会恢复的，没什么大碍 | 5 | 4 | 3 | 2 | 1 |
| 16 | 天气不好，先休息会儿看看 | 5 | 4 | 3 | 2 | 1 |
| 17 | 现在身边没有帮忙的人，还是等等再说吧 | 5 | 4 | 3 | 2 | 1 |
| 18 | 突然一侧肢体无力、沉重或麻木是最近累的 | 5 | 4 | 3 | 2 | 1 |
| 19 | 突然一侧或双眼视物模糊是用眼过度 | 5 | 4 | 3 | 2 | 1 |
| 20 | 晨起发现一侧肢体无力、笨拙，是睡觉受压了 | 5 | 4 | 3 | 2 | 1 |
| 21 | 突然的头痛和头晕，是感冒所致 | 5 | 4 | 3 | 2 | 1 |
| 22 | 出现症状首先想到的是休息观察 | 5 | 4 | 3 | 2 | 1 |
| 23 | 出现症状首先想到的是自行服药 | 5 | 4 | 3 | 2 | 1 |
| 24 | 症状不缓解（或加重）再去医院 | 5 | 4 | 3 | 2 | 1 |
| 25 | 叫救护车费用高，不叫救护车 | 5 | 4 | 3 | 2 | 1 |
| 26 | 第一时间想不到叫救护车 | 5 | 4 | 3 | 2 | 1 |
| 27 | 选择熟人介绍的中医医院 | 5 | 4 | 3 | 2 | 1 |

**Appendix2:The electronic ethic prove.**

**
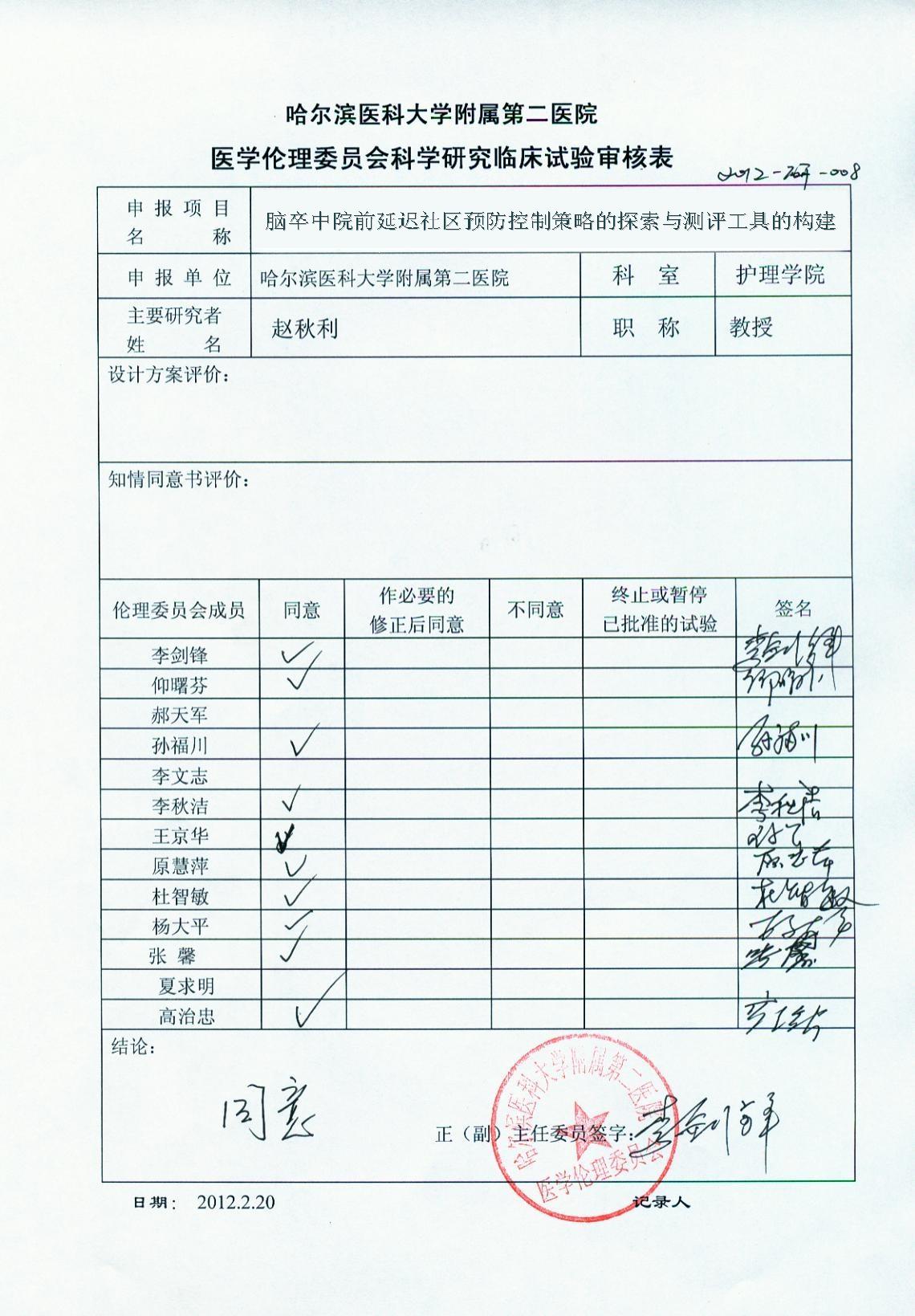
**
